# The *Mesobuthus martensii* genome reveals the molecular diversity of scorpion toxins

**DOI:** 10.1186/2045-3701-4-1

**Published:** 2014-01-03

**Authors:** Jianjie Ma, Yun-Bo Shi

**Affiliations:** 1 Department of Surgery, Davis Heart and Lung Research Institute, The Ohio State University Wexner Medical Center, Columbus, OH 43210, USA; 2 Section on Molecular Morphogenesis, Program in Cellular Regulation and Metabolism (PCRM), Eunice Kennedy Shriver National Institute of Child Health and Human Development (NICHD), National Institutes of Health (NIH), Bethesda, Maryland 20892, USA

**Keywords:** Scorpion, Mesobuthus martensii, Neurotoxin, Genome, Evolution

## Abstract

Recent complete sequencing of the genome of the Asian scorpion, *Mesobuthus martensii,* highlights the molecular diversity of its venom neurotoxin/defensin genes and interesting features of their evolution.

## 

Scorpion is a mysterious organism in the animal kingdom. It is a living fossil [1], has poisonous venom [2], and can be fluorescent [3]. Such unique features have increasingly attracted scientists’ attention and interests around the world. One exciting progress in scorpion research is a recent report from Dr. Wenxin Li’s group in China for the complete genome sequencing of the Asian scorpion, *Mesobuthus martensii*[4]. The *M. martensii* genome has an estimated size of 1323.73 Mb but interestingly contains predicted 32,016 genes, more than those in human [4]. The analysis by Cao et al. also revealed that *M. martensii* genome has, in addition to a large gene content, a high level of transposable element accumulation, a divergent evolution of venom neurotoxin/defensin genes, and an expansion of gene families often associated with unique biological features of scorpions [4].

One important finding from the genome sequence analysis is the molecular diversity of venom neurotoxins [4]. For the first time, Cao et al. systematically analyzed the gene structure and organization of neurotoxins at the genome level. They discovered 116 neurotoxin genes present in the *M. martensii* genome: 61 NaTx (toxins for sodium channels) genes, 46 KTx (toxins for potassium channels) genes, 5 ClTx (toxins for chloride channels) genes, and 4 CaTx (toxins for ryanodine receptors) genes. Compared to the known neurotoxins previously identified through cDNA cloning and biochemical purification, most of the 45 newly discovered neurotoxin genes belong to the NaTx and KTx families. Their analysis thus provided a different but complete picture of the molecular diversity of neurotoxins in *M. martensii*.

Earlier transcriptome analyses of scorpion venomous gland by Li’s group provided evidence that the scorpion venom has a large variety of biologically active peptides. The Li team began the scorpion research in 1990s by first constructing a cDNA library for the scorpion *M. martensii* in order to isolate and characterize new toxin genes [5]. Later, Ma et al. carried out a transcriptomic analysis of the venom gland of the scorpion *Scorpiops jendeki*[6]. Their work revealed that the venom of *Scorpiops jendeki* has more than 10 known types and 9 atypical types of peptides/proteins [6]. Subsequently, in 2010, they used both transcriptomic and proteomic analyses to determine the toxin content of the venom of the scorpion *Heterometrus petersii*[7]. In the same year, the Li group published a comparative analysis of the venom transcriptome of the scorpion *Lychas mucronatus* from two different geographical regions in China: one region in Hainan province and the other region in Yunnan province [8]. Interestingly, this study identified a large number of new venom molecules and also revealed that venom peptides/proteins of the same scorpion species from different geographical regions are highly diversified. These findings suggest the possibility that scorpions evolve in order to adapt to new environment by changing the primary structure and abundance of venom peptides/proteins. Last year, this same group continued their study with transcriptome analysis of three new scorpion species: two Buthidae species (*Lychas mucronatus* and *Isometrus maculatus*) and one Euscorpiidae species (*Scorpiops margerisonae*) [9]. More recently, a similar analysis of the venom glands from two scorpion species of the family Chaerilidae, *Chaerilus tricostatus* and *Chaerilus tryznai* revealed 14 types of venom peptides/proteins and 74 atypical venom molecules [10]. Their cumulative transcriptomic analyses were responsible for the majority of new toxin molecules discovered in the field of scorpion toxins. Together, these findings created the genetic resource libraries for scorpion toxin research and will help to accelerate the drug discovery of toxin peptides. In fact, by analyzing molecular diversity of scorpion toxins and their structure-function relationships, they were the first to reveal a critical role of acidic residue function in toxin activity [11] and developed a novel drug lead [12].

The analyses of the *M. martensii* genome by Cao et al. [4] revealed some unique features in the structure and organization of neurotoxin genes. Out of the 116 neurotoxin genes, 109 are expressed in the venomous gland. Most of the neurotoxin genes contain one intron located at the end of the coding region for the signal peptide, while a smaller number had no or two introns. 44% (51/116) of the neurotoxin genes are present in clusters on seventeen scaffolds. Within each cluster are neurotoxin genes of the same family that share similar gene structure and organization, indicating that frequent gene duplication occurred at the neurotoxin loci. Similar features of gene cluster and structural organization were also found for the six defensin genes in *M. martensii*, suggesting an evolutionary trajectory parallel to that of neurotoxin genes.

The *M. martensii* genome sequence also allowed Cao et al. [4] to investigate the origin of scorpion neurotoxin genes, a subject of intense recent debate. Hierarchical clustering was used to group the related neurotoxin genes from *M. martensii*, including 54 NaTx, 41 KTx, 5 ClTx and 6 defensins. Two major groups corresponding to pharmacological classes were formed. Group 1 comprises NaTx genes, whereas group 2 contains KTx, ClTx and defensin genes. These findings not only point to monophyly of the neurotoxin and definsin genes, but also implicate a strong structure-function relationship by the association of functional determinants with the sequence homology groups. They also suggest that NaTx likely diverged early from the common ancestor(s) of KTx, ClTx and defensin, and subsequently KTx, ClTx and defensins evolved into their separate families.

Together with the transcriptome analyses of the venomous glands from different Chinese scorpion species, the genome sequence of *M. martensii* allows the characterization of a large family of venom molecules, in addition to other genes involved in defense and detoxification of the animal species. Such information provides a valuable resource for further study on the biology of the venom toxin, as well as for future therapeutic approach that targets neurotoxins for treatment of human diseases. Clearly, more questions will arise from the completion of the *M. martensii* genome, such as those on the comparative genomics of scorpion toxins and the biological functions of scorpion toxins, which will attract new generation of scientists to work on this exciting field of research and development.

## Competing interests

The authors declare that they have no competing interests.

## Authors’ contributions

JM and YBS wrote the manuscript and approved the final manuscript.
